# Axillo-Femoral Bypass Followed by Pelvic Exenteration for Locally Advanced Sigmoid Colon Cancer Invading the Common Iliac Artery

**DOI:** 10.70352/scrj.cr.24-0001

**Published:** 2025-03-14

**Authors:** Moe Enari, Kay Uehara, Takeshi Yamada, Aitsariya Mongkhonsupphawan, Sho Kuriyama, Yasuyuki Yokoyama, Hiromichi Sonoda, Yuji Maruyama, Yosuke Ishii, Hiroshi Yoshida

**Affiliations:** 1 Department of Gastroenterological Surgery, Nippon Medical School, Tokyo, Japan; 2 Department of Cardiovascular Surgery, Nippon Medical School, Tokyo, Japan

**Keywords:** axillo-femoral bypass, total pelvic exenteration, conversion surgery

## Abstract

**INTRODUCTION:**

Initially, unresectable locally advanced colorectal cancers are still not uncommon. Despite recently developed systemic treatment has extended the survival of patients with unresectable and recurrent disease, surgical resection offers the chance for a cure or long-term survival. Recently, with improvement in the safety of major vascular reconstruction, several reports have suggested that extended pelvic tumor resection with vascular reconstruction with curative intent can be performed safely; however, the indications for arterial vascular reconstruction remain controversial and are reported with a literature review.

**CASE PRESENTATION:**

A 73-year-old male patient whose fever was greater than 40° was admitted to the emergency department of our hospital. Computed tomography (CT) revealed a large mass on the left side of the aortic bifurcation, and a diagnosis of unresectable sigmoid colon cancer was made (cT4bN1M0). The tumor had substantially invaded the iliopsoas muscle and intramuscular abscess, left hydronephrosis due to left ureteral invasion, invasion of the left common and external iliac artery, and congestive edema of the left leg were observed. Transverse colostomy and left nephrostomy were created and percutaneous drainage of the iliopsoas abscess was performed. Four cycles of FOLFOX + bevacizumab were administered after the systemic infection had resolved. The tumor volume decreased, and no new lesions were observed. The patient underwent left axillo-femoral bypass followed by total pelvic exenteration, combined left common and external iliac artery resection, and right ureterocutaneostomy. His postoperative course was uneventful. Pathology revealed ypT4b (bladder) N0M0, ypStage II disease. The patient was followed without adjuvant chemotherapy and had no recurrence as of 10 months after surgery.

**CONCLUSIONS:**

We experienced a case of total pelvic exenteration combined with the common and external iliac artery and reconstruction via axillo-femoral bypass. When treating complicated cases that cannot be cured by a single operation, it is necessary to carefully consider the optimal pathway for radical resection and to be very familiar with perioperative treatment and reconstructive methods.

## INTRODUCTION

With advances in screening and imaging diagnosis, the number of colorectal cancer cases that are already highly advanced at initial presentation has decreased^1)^; however, initially unresectable locally advanced colorectal cancers are still not uncommon. While recently developed systemic treatment has extended the survival of patients with unresectable and recurrent diseases, surgical resection offers the chance for a cure or long-term survival. The survival time of patients who undergo surgical resection is significantly longer, by years, than that of patients who receive systemic treatment alone.^2)^ However, complicated pelvic surgeries still have a high complication rate and should be performed at restricted institutions. We experienced a case of total pelvic exenteration combined with the common and external iliac artery and reconstruction via axillo-femoral (A-F) bypass, which was previously considered a contraindication for surgery at our institution in principle.^3)^

## CASE PRESENTATION

**Fig. 1:**
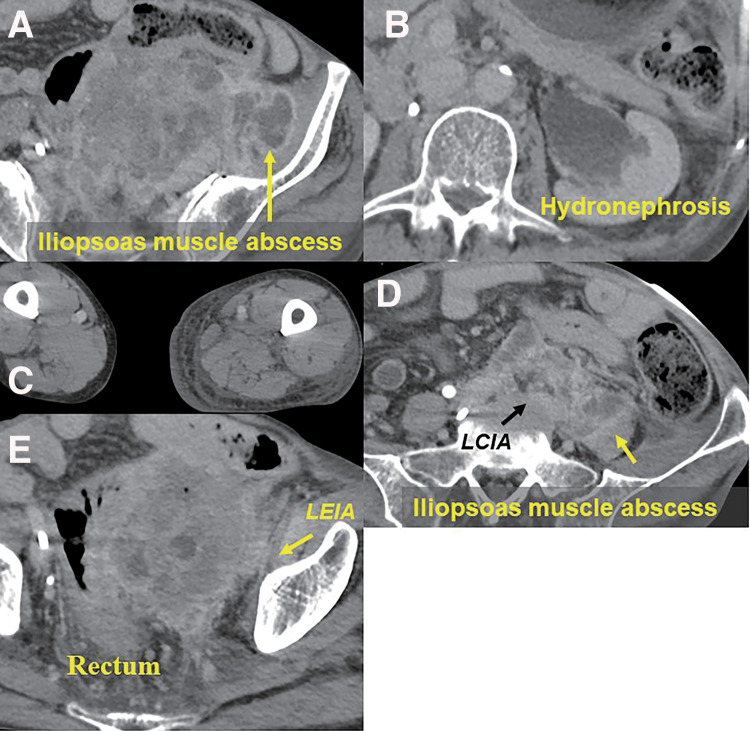
Initial CT revealed an initially unresectable sigmoid colon cancer. The tumor had invaded the iliopsoas muscle and was accompanied by intramuscular abscess formation (**A**), left hydronephrosis due to left ureteral invasion (**B**), urinary bladder invasion, and congestive edema of the left lower leg (**C**) due to invasion of the common to the external iliac artery/vein (**D** and **E**). CT; computed tomography, LEIA; left external iliac artery, LCIA; left common iliac artery

A 73-year-old male patient with a fever of more than 40° and difficulty in physical movement was admitted to the emergency department of our hospital. The patient had alcoholic cirrhosis of the liver. The laboratory findings revealed a strong inflammatory response, including a white blood cell count of 27200/μL, a C-reactive protein level of 12.85 mg/dL, and a high blood ammonia level of over 500 μg/dL. The tumor marker was high, with a carcinoembryonic antigen (CEA) level of 62.4 ng/mL. Computed tomography (CT) revealed a large mass near the aortic bifurcation. The tumor was initially diagnosed as unresectable sigmoid colon cancer because of iliopsoas muscle invasion and intramuscular abscess formation, left hydronephrosis due to left ureteral invasion, urinary bladder invasion, and congestive edema of the left lower leg due to invasion of the common to external iliac artery/vein (**Fig. 1**). Colonoscopy revealed a circumferential lesion at 15 cm from the anal verge, and biopsy demonstrated that the tumor was a well-differentiated tubular adenocarcinoma. Genetic testing revealed that the tumor was a RAS mutated, BRAF wild type, and microsatellite stability tumor.

**Fig. 2:**
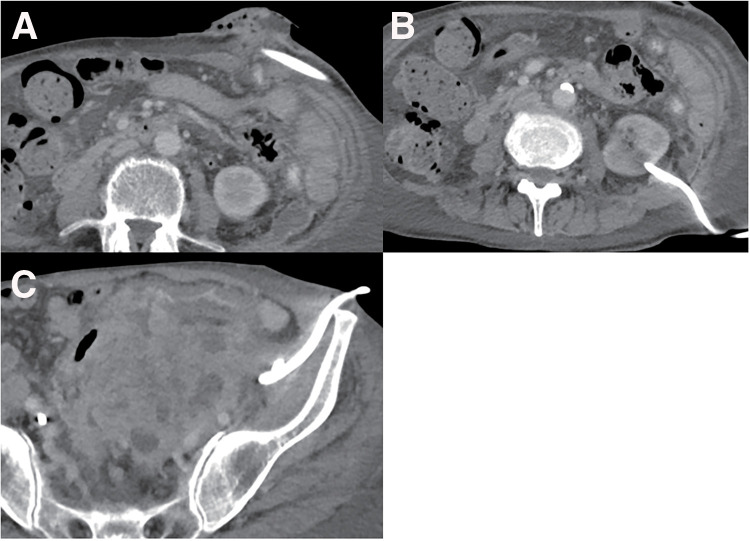
A transverse colostomy was performed (**A**), then a left nephrostomy was created for left hydronephrosis (**B**), and percutaneous drainage was performed for the iliopsoas abscess (**C**).

**Fig. 3:**
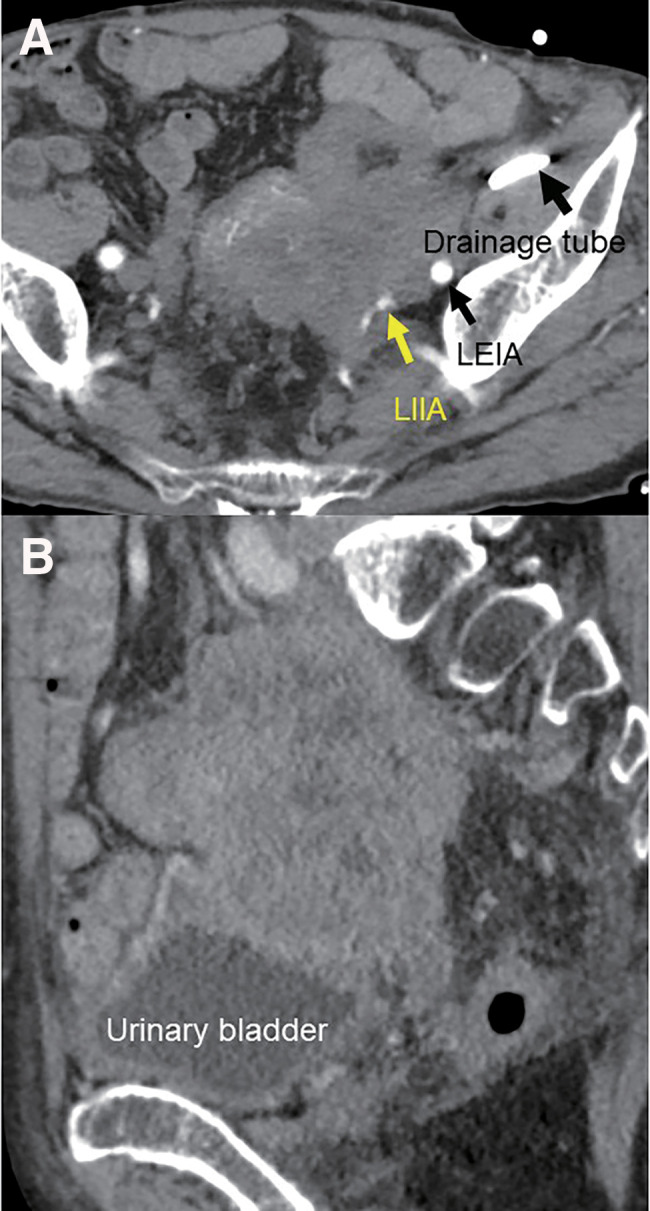
The tumor volume decreased after chemotherapy; however, invasion of the bladder (**A**) and common and external iliac vessels (**B**) remained. LIIA; left internal iliac artery, LEIA; left external iliac artery

**Fig. 4:**
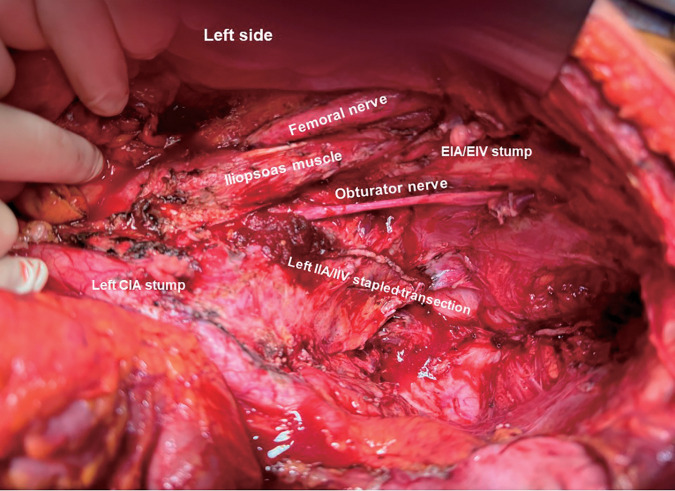
Intraoperative photography after total pelvic exenteration, combined resection of the left common and external iliac vessels, and partial resection of the iliopsoas muscle. CIA; common iliac artery, IIA; internal iliac artery, IIV; internal iliac vein, EIA; external iliac artery, EIV; external iliac vein

First, a transverse colostomy was performed; then, a left nephrostomy was created for left hydronephrosis, and percutaneous drainage was performed for iliopsoas abscess, resulting in an improved inflammatory response (**Fig. 2**). After 4 courses of FOLFOX + bevacizumab therapy, the tumor volume decreased, and no new lesions appeared (**Fig. 3**), and the CEA level decreased to 10.1 ng/mL. Four months after the initial visit, it was determined that an R0 resection was possible via total pelvic exenteration with iliac artery reconstruction. The patient underwent left A-F bypass using an expanded polytetrafluoroethylene (e-PTFE) graft (8 mm) with ligation of the proximal common femoral artery followed by total pelvic exenteration and combined resection of the left common and external iliac artery/vein (**Fig. 4**) and right ureterocutaneostomy. The distal rectum was transected, and the anus was preserved; however, colorectal anastomosis was avoided. The operative time was 13 hours 39 minutes, blood loss was 3276 mL, and transfusions of red blood cells (8 units) and fresh frozen plasma (6 units) were intraoperatively performed.

**Fig. 5:**
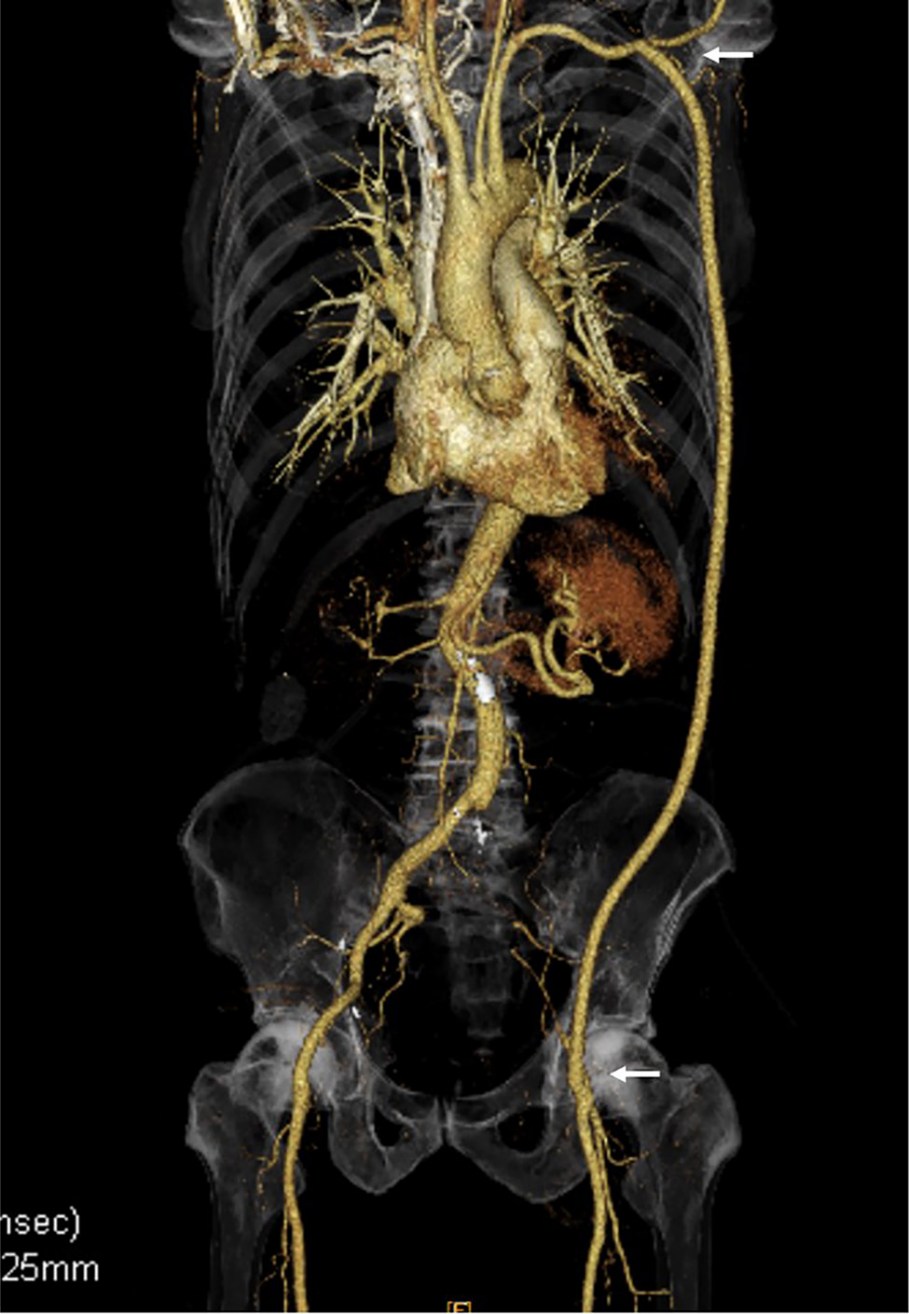
CT on postoperative day 7 revealed no issue at all, with patency of the axillo-femoral bypass.

Pathological findings revealed that the tumor was ypT4b (bladder) N0M0, ypStage II. Treatment of left leg edema and gait rehabilitation took time, but no postoperative complications of Clavien-Dindo grade 2 or higher were observed (**Fig. 5**), and the patient was discharged on the 44^th^ day after surgery. No adjuvant chemotherapy was administered due to performance status (PS) of 2. The patient was alive 9 months after surgery and had not experienced recurrence and rebound of left leg edema.

## DISCUSSION

It is not uncommon to encounter colorectal cancer patients with aggressive local extension but without distant metastasis. Even today, with the development of systemic therapy, these patients are the ones who can benefit the most from the surgical approach. The patient was in poor general condition and had highly locally advanced cancer at the time of initial transport, but the absence of distant metastasis led to a thorough discussion on how to accomplish curative resection. The tumor was initially diagnosed as unresectable because of its widespread invasion into the iliopsoas muscle to form an abscess, extensive involvement of the left common and external iliac artery/vein, and massive invasion into the bladder. The only surgical options for a cure were total pelvic exenteration (TPE) and vascular resection/reconstruction, and to make this possible, it is necessary to improve the patient’s general condition and decrease the tumor volume with systemic therapy.

The first step was to resolve inflammation. Stoma creation, nephrostomy, and percutaneous drainage were performed; however, these procedures should be performed to consider the patient’s future. It is usually necessary to carefully consider the promising site of the intestinal tract and abdominal wall that is suitable for stoma creation. Recreating a stoma should be avoided, increasing the risk of overall surgical site infection (SSI)^4)^ and often causing scarring of the skin, making it difficult to manage with a new stoma and decreasing PS.^5)^ In principle, stoma creation on the right abdominal wall should be considered a contraindication, when urinary tract reconstruction may be necessary in the future since the ileal conduit is usually created on the right side. Occasionally, a loop ileostomy is created on the right side, which is an option for later use as an ileal conduit; however, it must be created with the oral side facing in a caudal direction. Furthermore, it should be considered that it is not useful for diverting colon contents; thus, its indication should be strictly considered. In this case, since nephrostomy was created on the left side, the final goal was determined to have either ileal conduit after TPE or right ureterocutaneostomy, and since drainage of the oral side of the sigmoid colon cancer was urgently needed, a stoma was created on the left upper abdomen using not left-sided but mid-transverse colon. In the last surgery, the anal side of the stoma can be divided just below the abdominal wall and used as a permanent colostomy, or it also can be used as a diverting stoma after anastomosis when preserving the anus. There may be arguments against the fact that urinary tract reconstruction and anal preservation were not performed; however, we believe that in surgeries that involve considerable risk, safety should be prioritized and the surgery should be simplified.

The discussion focused mostly on vascular reconstruction. Conventionally, advanced or recurrent colorectal cancer requiring major vascular reconstruction has often been considered a relative contraindication for curative resection.^3)^ However, recently, with improvement in the safety of major vascular reconstruction, several reports have suggested that extended pelvic tumor resection with vascular reconstruction with curative intent can be performed safely.^6–8)^ A recent narrative review reported that extended pelvic surgery with major vessel reconstruction has equivalent results in R0 resection rate, complication rate, and survival compared with that without vascular reconstruction, although the operative time was longer and blood loss was significant.

For veins, whether they should be ligated or reconstructed is still controversial because when performing vein reconstruction, the risk of thrombosis is a major problem. Non-reconstruction without risk of deep venous thrombosis can be an option, although the lower leg edema can be severe.^6)^ In addition, in the case of advanced or recurrent colorectal cancer, anti-angiogenic agents are often used; however, venous thrombosis induced by anti-angiogenic agents is a very problematic adverse effect that sometimes makes it impossible to continue using the drug. It is thought that venous turbulence after vein reconstruction may rather promote thrombus formation, and from this perspective, I believe that vein reconstruction is not necessary for surgery for advanced or recurrent colorectal cancer. In this case, the left external iliac vein was obstructed due to tumor invasion at the time of the initial diagnosis, and edema of the left lower leg was remarkable. However, at the time of surgery, although it was unclear from the images, it had almost disappeared due to the development of collateral blood vessel pathways via a route outside the pelvis. Although it was necessary to perform a combined resection of the left common and external iliac artery/vein, the postoperative edema in the left lower legs was mild, and there has been no rebound of edema even 9 months after the surgery.

On the other hand, arterial vascular reconstruction requires certainty of success to avoid leg ischemia. The effectiveness of autologous vein grafts, such as saphenous veins, has been reported when resection is limited,^9)^ but these grafts are often thin and limited in length, making them unsuitable for longer arterial reconstruction. Allogeneic biologic grafts can be used in infected surgical fields and are advantageous because long-term anticoagulation treatment is not needed^10)^; however, the major problem in Japan is that allogeneic biologic materials are not covered by national insurance for pelvic surgery. However, it is not possible to perform vascular reconstruction through an infected surgical field after intestinal resection with synthetic grafts. On the other hand, femoro-femoral (F-F) bypass and A-F bypass are used as reconstruction options via an extraanatomical route.^11,12)^ F-F bypass is the preferred procedure for bypass in leg vascular disease because of the greater distance of A-F bypass, and the superior postoperative patency rate of F-F bypass.^13,14)^ However, the data are from patients with leg vascular disease, and there are not any data from patients with healthy leg vessels who underwent complicated resection for pelvic tumors; thus, these findings do not support the conclusion that A-F bypass is not a viable option. In addition, it is important to safely perform extended pelvic surgery with a fine view; thus, skin incisions often extend into the pubic symphysis. In such cases, the F-F bypass is exposed or closed to the surgical field and has the potential to be harmed by a postoperative SSI. In A-F bypass, the artificial vessel passes outside of the abdominal wall, allowing the surgeon to concentrate on safe resection without worrying about the artificial vessel during the operation, which is our preferred choice for vascular reconstruction in pelvic surgery. Attention should be given to ligating the femoral artery on the proximal side of the bypass, which, if not done, can lead to arterial competition resulting in thrombus formation.

## CONCLUSION

When treating complicated cases that cannot be cured by a single operation, it is necessary to carefully consider the optimal pathway for radical resection and to be very familiar with perioperative treatment and reconstructive methods.

## DECLARATIONS

### Funding

The authors declare that this case report did not receive any funding.

### Authors’ contributions

The draft of the manuscript was written by ME and KU, TY, YM, and YI revised the manuscript.

All authors commented on previous versions of the manuscript and approved the final manuscript.

Axillo-femoral bypass followed by pelvic exenteration for locally advanced sigmoid colon cancer invading the common iliac artery by Kay Uehara is licensed under CC BY-NC-ND 4.0.

### Availability of data and materials

Data sharing is not applicable to this article, as no datasets were generated or analyzed.

### Ethics approval and consent to participate

Not applicable.

### Competing interests

The authors declare that they have no competing interests.
